# Supplementary material for: Preoperative Classification of Peripheral Nerve Sheath Tumors on MRI Using Radiomics
Source: Cancers (Basel). 2024 May 28;16(11):2039. doi: 10.3390/cancers16112039 (PMC11170987; doi:10.3390/cancers16112039)

Supplementary Tables

Table S1: *Rule-based scoring of tumor segmentation based on visual inspection by the user.* [39]

Segmentation score	Definition
Excellent	The segmentation is perfectly aligned with the tumor and requires no adjustments. For this score, the segmentation volume should overlap with the tumor for at least 95%.
Sufficient	The segmentation is aligned with the tumor, however, could benefit from minor adjustments. For this score, the segmentation volume should overlap with the tumor for at least 75%.
Insufficient	The segmentation misses parts of the tumor, or parts are overlapping with normal tissue, therefore major adjustments are required. For this score, the segmentation volume should overlap with the tumor for at least 50%.
Incorrect	the segmentation is not overlapping with the tumor, or missing large areas of the tumor. For this score, the segmentation volume does not overlap with the tumor for <50%.

If the tumor cannot be located in the image, the clinician can score as "Cannot locate tumor".

Table S2: *Overview of included MRI sequences and several acquisition parameters.*

MRI sequence	T1w	T2w	T1w-FS-GD	T1w-SPIR-GD	T2w-FS	T2w-STIR
Available number of scans	90	66	39	12	41	28
Slice Thickness (mm)*	4.9 ± 2.8	4.7 ± 3.4	4.8 ± 1.4	4.3 ± 1.0	5.4 ± 2.0	4.5 ± 1.3
Repetition time (ms)*	620 ± 435	3756 ± 2667	657 ± 687	516 ± 187	4831 ± 2574	4016 ± 2187
Echo time (ms)*	12.4 ± 3.8	79.0 ± 38.1	11.6 ± 5.5	14.3 ± 5.2	80.5 ± 22.6	59.5 ± 32.5

Abbreviations: FS, Fat Saturation; SPIR, Spectral Presaturation with Inversion Recovery; GD, Gadolinium Contrast; STIR, Short τ Inversion Recovery.

* Values are mean ± standard deviation

Table S3. Overview of the 564 features used in this study, adopted from Vos et al. (2019) [24]. GLCM features were calculated in four different directions (0, 45, 90, 135 degrees) using 16 gray levels and pixel distances of 1 and 3. LBP features were calculated using the following three parameter combinations: 1 pixel radius and 8 neighbors, 2 pixel radius and 12 neighbors, and 3 pixel radius and 16 neighbors. Gabor features were calculated using three different frequencies (0.05, 0.2, 0.5) and four different angles (0, 45, 90, 135 degrees). LoG features were calculated using three different widths of the Gaussian (1, 5 and 10 pixels). Vessel features were calculated using the full mask, the edge, and the inner region. Local phase features were calculated on the monogenic phase, phase congruency and phase symmetry.

**Histogram (13 features)**	**LoG (13*3=39 features)**	**Vessel (12*3=39 features)**	**Orientation (9 features)**	**Local phase (13*3=39 features)**	**GLCM (MS) (6*3*4*2=144 features)**	**Gabor (13*4*3=156 features)**	**NGTDM (5 features)**	**LBP (13*3=39 features)**
min<br>max<br>mean<br>median<br>std<br>skewness<br>kurtosis<br>peak<br>peak position<br>range<br>energy<br>quartile range<br>entropy	min<br>max<br>mean<br>median<br>std<br>skewness<br>kurtosis<br>peak<br>peak position<br>range<br>energy<br>quartile<br>entropy	min<br>max<br>mean<br>median<br>std<br>skewness<br>kurtosis<br>peak<br>peak position<br>range<br>energy<br>quartile<br>entropy	theta_x<br>theta_y<br>theta_z<br>COM index x<br>COM index y<br>COM index z<br>COM x<br>COM y<br>COM z	min<br>max<br>mean<br>median<br>std<br>skewness<br>kurtosis<br>peak<br>peak position<br>range<br>energy<br>quartile<br>entropy	contrast (normal, MS mean + std)<br>dissimilarity (normal, MS mean + std)<br>homogeneity(normal, MS mean + std)<br>angular second moment (ASM) (normal, MS mean + std)<br>energy (normal, MS mean + std)<br>correlation (normal, MS mean + std)	min<br>max<br>mean<br>median<br>std<br>skewness<br>kurtosis<br>peak<br>peak position<br>range<br>energy<br>quartile range<br>entropy	busyness<br>coarseness<br>complexity<br>contrast<br>strength	min<br>max<br>mean<br>median<br>std<br>skewness<br>kurtosis<br>peak<br>peak position<br>range<br>energy<br>quartile range<br>entropy

**GLSZM (16 features)**	**GLRM (16 features)**	**GLDM (14 features)**	**Shape (35 features)**
Gray Level Non Uniformity<br>Gray Level Non Uniformity Normalized<br>Gray Level Variance<br>High Gray Level Zone Emphasis<br>Large Area Emphasis<br>Large Area High Gray Level Emphasis<br>Large Area Low Gray Level Emphasis<br>Low Gray Level Zone Emphasis<br>SizeZoneNonUniformity<br>SizeZoneNonUniformityNormalized<br>SmallAreaEmphasis<br>SmallAreaHighGrayLevelEmphasis<br>SmallAreaLowGrayLevelEmphasis<br>ZoneEntropy<br>ZonePercentage<br>ZoneVariance	Gray Level Non Uniformity<br>Gray Level Non Uniformity Normalized<br>Gray Level Variance<br>High Gray Level Run Emphasis<br>Long Run Emphasis<br>Long Run High Gray Level Emphasis<br>Long Run Low Gray Level Emphasis<br>Low Gray Level Run Emphasis<br>RunEntropy<br>RunLengthNonUniformity<br>RunLengthNonUniformityNormalized<br>RunPercentage<br>RunVariance<br>ShortRunEmphasis<br>ShortRunHighGrayLevelEmphasis<br>ShortRunLowGrayLevelEmphasis	Dependence Entropy<br>Dependence Non-Uniformity<br>Dependence Non-Uniformity Normalized<br>Dependence Variance<br>Gray Level Non-Uniformity<br>Gray Level Variance<br>High Gray Level Emphasis<br>Large Dependence Emphasis<br>Large Dependence High Gray Level Emphasis<br>Large Dependence Low Gray Level Emphasis<br>Low Gray Level Emphasis<br>Small Dependence Emphasis<br>Small Dependence High Gray Level Emphasis<br>Small Dependence Low Gray Level Emphasis	compactness (mean + std)<br>radial distance (mean + std)<br>roughness (mean + std)<br>convexity (mean + std)<br>circular variance (mean + std)<br>principal axes ratio (mean + std)<br>elliptic variance (mean + std)<br>solidity (mean + std)<br>area (mean, std, min + max<br>volume (total, mesh, volume)<br>elongation<br>flatness<br>least axis length<br>major axis length<br>minor axis length<br>maximum diameter 3D<br>maximum diameter 2D (rows, columns, slices)<br>sphericity<br>surface area<br>surface volume ratio

Abbreviations: COM: center of mass; GLCM: gray level co-occurrence matrix; MS: multi slice; NGTDM: neighborhood gray tone difference matrix; GLSZM: gray level size zone matrix; GLRLM: gray level run length matrix; LBP: local binary patterns; LoG: Laplacian of Gaussian; std: standard deviation.

Table S4: *Overview of the changes between two assessments by radiologists.*

Radiologist	Total number of changes	From benign to malignant		From malignant to benign	
		New errors	Corrections	New errors	Corrections
Radiologist 1	11	7	2	2	0
Radiologist 2	6	3	1	1	1

The differences were observed between the two assessments by radiologists. During the first assessment, radiologists had access only to MRI scans of tumors, whereas in the second assessment, they had access to both MRI scans and clinical features of tumors. "New errors" denote changes resulting in incorrect results in the second assessment, while "corrections" refer to changes leading to accurate results in the second assessment.

<u>Supplementary Figures</u>

Figure S1: Visualization of the nested cross-validation for the evaluation, adopted from Vos et al. (2019) [24]. The inner fivefold random-split cross--validation was conducted solely on the training set to find the robust ensemble which averaged the predictions from 100 top performing models. The test set was used only for the evaluation of the final models. The performance was computed based on 100 times of random-split cross--validation. The dataset was split in a stratified manner to ensure the balance between MPNST and BPNST.

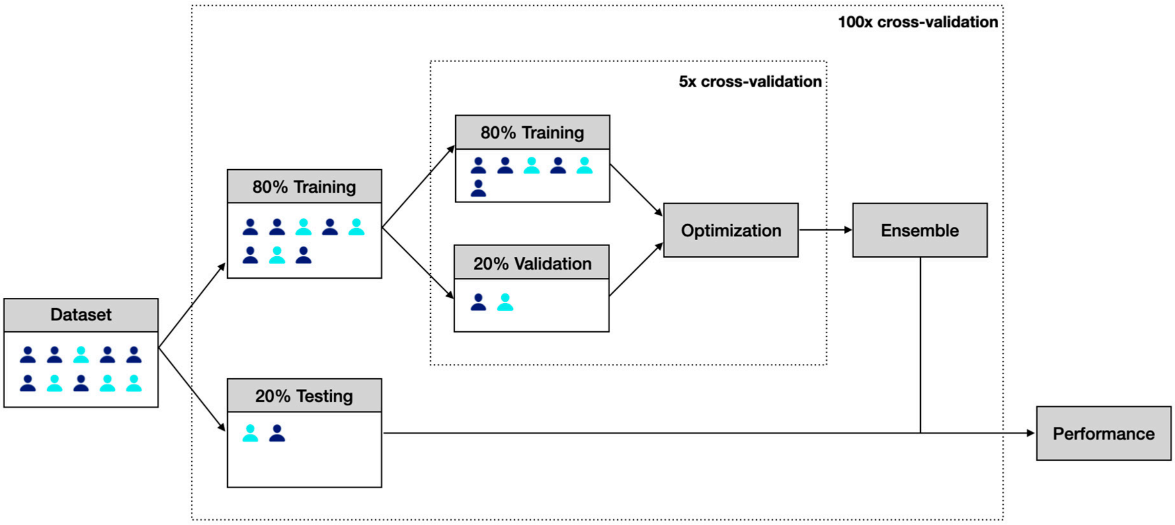

Figure S2: Univariate statistical testing of 564 radiomics features employing the Mann-Whitney U test. Colors correspond to the different feature groups. The magenta dotted red lines indicate the thresholds of p-values with Bonferroni correction for multiple testing (9x10^{-5}) and without (0.05). For brevity, only features with p-value below 9x10^{-5} are presented with names.

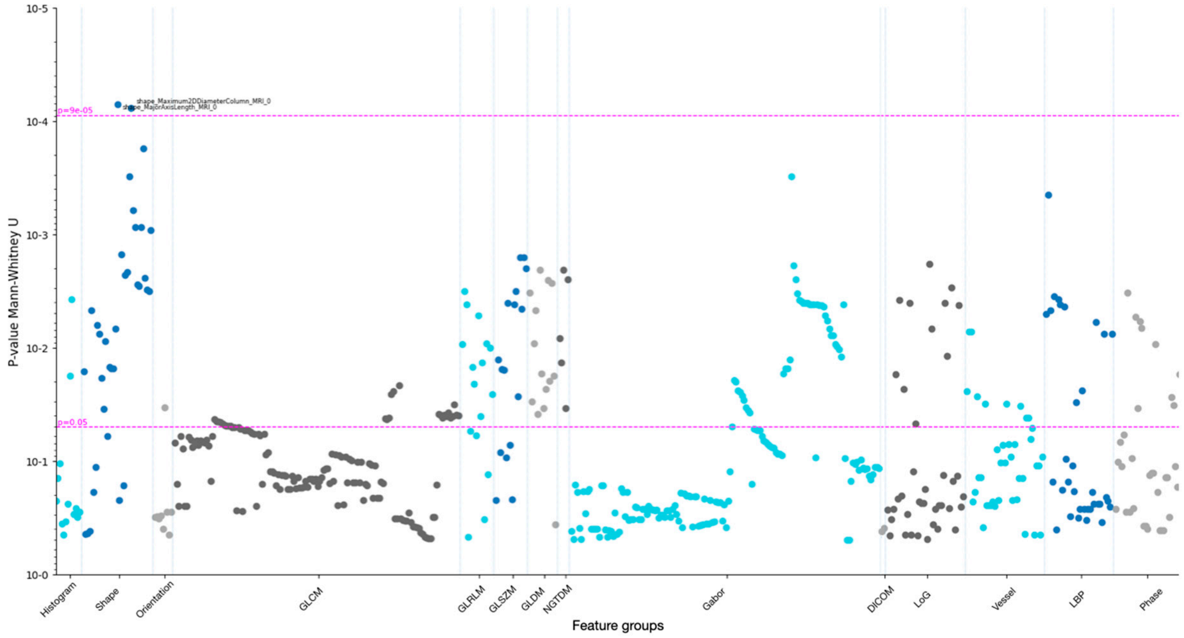

Supplement: Supplementary file 1 [file cancers-16-02039-s001.zip › cancers-3006356-supplementary.pdf]
